# How Do Transposable Elements Activate Expression of Transcriptionally Silent Antibiotic Resistance Genes?

**DOI:** 10.3390/ijms23158063

**Published:** 2022-07-22

**Authors:** Aleksander Lipszyc, Magdalena Szuplewska, Dariusz Bartosik

**Affiliations:** Department of Bacterial Genetics, Institute of Microbiology, Faculty of Biology, University of Warsaw, Miecznikowa 1, 02-096 Warsaw, Poland; a.lipszyc2@student.uw.edu.pl (A.L.); m.szuplewska@uw.edu.pl (M.S.)

**Keywords:** insertion sequence, transposon, transposable elements, antibiotic resistance, antibiotic resistance determinants, gene expression, transcriptionally silent genes, gene activation, promoter delivery

## Abstract

The rapidly emerging phenomenon of antibiotic resistance threatens to substantially reduce the efficacy of available antibacterial therapies. Dissemination of resistance, even between phylogenetically distant bacterial species, is mediated mainly by mobile genetic elements, considered to be natural vectors of horizontal gene transfer. Transposable elements (TEs) play a major role in this process—due to their highly recombinogenic nature they can mobilize adjacent genes and can introduce them into the pool of mobile DNA. Studies investigating this phenomenon usually focus on the genetic load of transposons and the molecular basis of their mobility. However, genes introduced into evolutionarily distant hosts are not necessarily expressed. As a result, bacterial genomes contain a reservoir of transcriptionally silent genetic information that can be activated by various transposon-related recombination events. The TEs themselves along with processes associated with their transposition can introduce promoters into random genomic locations. Thus, similarly to integrons, they have the potential to convert dormant genes into fully functional antibiotic resistance determinants. In this review, we describe the genetic basis of such events and by extension the mechanisms promoting the emergence of new drug-resistant bacterial strains.

## 1. Introduction

The extensive use of antibiotics facilitates the selection of highly resistant bacterial strains or variants, which are being isolated with increasing frequency from different environments, including water and soil [1]. The spread of such microorganisms, referred to as “antibiotic resistance (AR) pollution”, has been identified by the World Health Organization (WHO) as one of the major threats to global public health [2], resulting in a significant reduction in the effectiveness of available antibacterial therapies. Today, over 30 different classes of antibiotics (natural or synthetic) are in clinical use, including: β-lactams (carbapenems, cephalosporins, monobactams, and penicillin), aminoglycosides, amphenicols, macrolides, polypeptides, sulfonamides, and tetracyclines [3]. Resistance mechanisms developed by bacteria have been identified against representatives of all the antibiotic classes [1]. Understanding the molecular basis of mechanisms leading to the emergence of new resistant strains and increasing knowledge of the direction and dynamics of resistance determinant dissemination are therefore invaluable. 

The physiological and genetic bases of AR can vary widely. Resistance mechanisms fall into two groups: intrinsic (innate) and extrinsic (acquired). Intrinsic AR to a certain antibiotic or a group of related antibiotics is usually characteristic for all strains of a given bacterial species or genus. This kind of resistance may result from the inability of an antibiotic to reach its target, a lack of affinity for the target, the presence of multi-substrate efflux pumps, or some other chromosomally determined resistance mechanism [4]. A much more important evolutionary role is attributed to extrinsic resistance, which may arise from the mutation of a particular gene or acquisition of AR determinants from other, even phylogenetically distant, microorganisms via horizontal gene transfer (HGT) [5]. In this case, AR phenotypes arise in bacterial cells that were previously susceptible to a given compound. Therefore, such resistance may emerge in subpopulations of a particular bacterial species.

A very important role in horizontal transmission of AR genes is attributed to mobile genetic elements (MGEs), primarily transposable elements (TEs), but also plasmids and integrative conjugative or mobilizable elements (ICEs and IMEs), effective vehicles that facilitate and accelerate gene flux among bacteria [6]. TEs encode transposase (TPase), enzymes that catalyze the process of transposition, i.e., illegitimate recombination within one or between two DNA molecules at different, usually random, genomic locations [7]. Notably, TPase genes are the most prevalent genes in sequenced bacterial genomes and microbial metagenomes, which indicates that these recombinases significantly affect the structure and function of bacterial genomes [8]. In addition, they can promote diverse genetic rearrangements (insertions, deletions, inversions, duplications, and translocations) as well as the generation of transient cointegrate replicons, which stimulate the exchange of genes between different DNA molecules co-residing in a cell [9]. Moreover, due to their highly recombinogenic nature, TEs can mobilize chromosomal genes (including AR genes) for transposition and can introduce them, as an integral part of newly generated larger transposable units, into the pool of mobile DNA involved in HGT. A plethora of transposons (Tns) containing diverse AR genes have been identified in the nucleotide sequences of bacterial chromosomes and plasmids, thus, corroborating the role of TEs in the dissemination of resistance phenotypes [10,11,12]. An equally important feature of many TEs is their ability to provide (or generate) promoters that can drive or modulate the expression of genes located adjacent to the TE insertion site [13]. This phenomenon is particularly important given the relatively frequent occurrence of transcriptionally silent genes (including AR genes) in bacterial genomes.

## 2. Silent AR Genes 

By definition, silent (or cryptic) genes are those that are not expressed or are expressed very poorly under any conditions. The concept of silent genes and their significance have been reviewed recently by [14]. One of several mechanisms may be responsible for the lack of gene expression, including mutations in key DNA regions responsible for transcription initiation or the negative side effects of a cellular gene regulatory circuit, for example, the constitutive activity of a strong negative transcriptional regulator [14]. It is also important to emphasize that genes acquired from evolutionarily distant bacteria by HGT may contain promoters that are not recognized by the transcriptional machinery of the new host. However, even fully functional genes can be selectively silenced by cellular factors, including histone-like nucleoid-structuring (H-NS) proteins, which specifically target and silence horizontally acquired AT-rich DNAs (xenogeneic silencing) [15,16]. 

The occurrence of silent AR genes in bacterial genomes has been established by numerous studies [14], but little is known about their distribution and prevalence. These unexpressed genes are usually identified through large-scale screening, employing both phenotypic (minimal inhibitory concentration determination and disc diffusion tests) and genotypic assays (increasingly by whole genome-sequencing, WGS). The number of strains that are susceptible to a given antibiotic and also carry a resistance determinant to that compound varies depending on the type of resistance and the microbial community tested. Some studies have identified individual strains with silent AR genes [17,18,19], while their number was surprisingly high in others. For example, Jiang et al. [20] showed that nearly 80% (69 out of 87) of *Vibrio parahaemolyticus* strains isolated from cultured sea cucumbers (*Apostichopus japonicus*), carried a silent sulfonamide (folic acid synthesis inhibiting antibiotic) resistance *sul2* gene. In an analysis of multidrug-resistant *Klebsiella pneumoniae* strains responsible for an infection outbreak in Taiwan, 25% (10 out of 40) carried non-expressed imipenem-type carbapenem (cell wall synthesis inhibiting antibiotic) resistance genes [21]. A similar percentage of strains containing silent *catA1* chloramphenicol (ribosome-targeting antibiotic) resistance genes was observed in a pool of seafood-associated non-typhoidal *Salmonella* strains isolated on the southwest coast of India [19]. 

There is no doubt that the analysis of AR based solely on phenotypic testing provides an incomplete picture of the resistance potential of bacterial populations. Of particular concern is the fact that dormant AR genes are able to recover their expression quite easily. Much discussion on this phenomenon has focused on integron/superintegron promoterless AR gene cassettes (i.e., non-autonomous integrative mobile genetic elements), which can be converted into functional fully expressed genes upon their incorporation into integrons by site-specific recombination catalyzed by tyrosine recombinases [22]. This review expands this picture by examining the role of TEs and the process of transposition in the activation of silent AR genes. We describe the genetic basis of such events and, by extension, the mechanisms promoting the emergence of new drug-resistant bacterial strains. Given the prevalence of these elements in bacterial genomes and their considerable recombinogenic potential, it is likely that they play a key role in activating silent genes. 

## 3. Classification and General Features of TEs

TEs likely derive from transposable temperate phages. They are highly diverse in terms of their structure, specific properties, and mode of transposition. These characteristics have been thoroughly discussed in several excellent reviews [13,23,24,25,26,27,28]. Nevertheless, for a better understanding of the phenomena and mechanisms described below, some basic information on TEs is provided here. 

**Figure 1:**
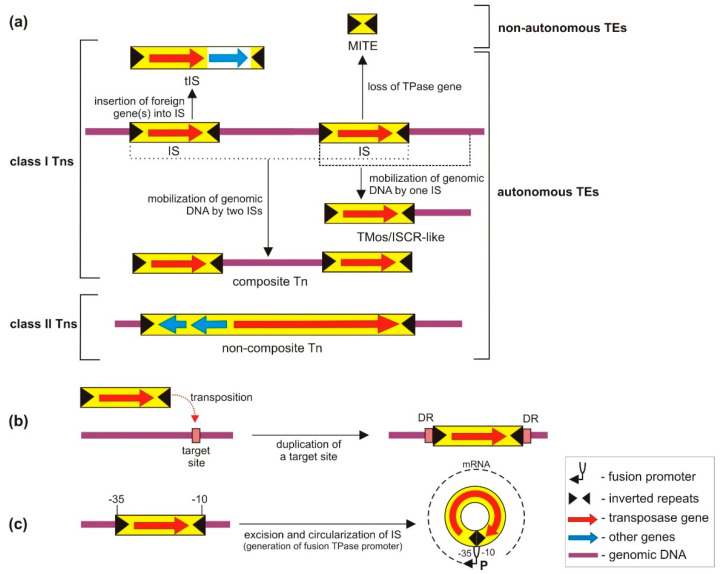
The main structural types and specific properties of bacterial TEs: (**a**) Simplified view of the structural diversity of class I and class II transposons. IS—insertion sequence; tIS—transporter IS; MITE—miniature inverted-repeat transposable element. The class II Tns are represented here by a Tn*3* family element; (**b**) duplication of a target site during transposition and generation of directly repeated sequences (DRs) flanking the inserted TE; (**c**) divided promoter of a TPase gene. After IS circularization, the −10 and −35 hexamers form a strong fusion promoter (P) enabling efficient TPase expression.

The simplest TEs are insertion sequences (ISs) that carry only genes related to transposition and regulation of this process. Most ISs contain only one or two open reading frames (ORFs) encoding the TPase, and short (10–40 bp) terminal inverted repeat sequences (IRs; IRL—left IR, IRR—right IR) where the TPase specifically binds and initiates further steps of transposition (Figure 1a). ISs and IS-dependent elements are classified as class I transposons [24]. The IS-driven TEs include (a) composite transposons composed of two ISs and a mobilized genomic DNA segment in between, (b) TMos/ISCR-like elements carrying a single IS and an adjacent fragment of genomic DNA mobilized by the IS [29,30,31], and (c) transporter ISs (tISs), IS derivatives carrying passenger genes with different functions [26] (Figure 1a). Another major group of TEs are class II transposons (also known as unit or non-composite transposons), represented mainly by the Tn*3*-family elements. Unlike composite Tns, the transposition of class II transposons is not dependent on ISs. They contain terminal IRs and accessory genes that are integral parts of the element [32]. It is also important to mention non-autonomous TEs (e.g., MITEs—miniature inverted-repeat transposable elements; Figure 1a), which do not encode their own TPase, and therefore, their mobility is fully dependent on the enzymatic activity of compatible TPases encoded in trans by functional autonomous elements [33].

The vast majority of TEs transpose into random genomic locations and this process is usually accompanied by duplication of a short target sequence (DR; direct repeats, flanking inserted TE), whose length is specific to a particular group of related elements [24] (Figure 1b). Transposition may proceed via either a conservative (translocation of an element to another genomic location) or replicative mode (translocation of a duplicated copy of an element). There are two modes of replicative transpositions: target-primed and donor-primed modes. The target-primed mode involves formation of a Shapiro intermediate that is converted to a cointegrate by DNA replication, and the cointegrate is, in turn, resolved to a donor molecule and target sequence carrying the new insertion [34]. The donor-primed mode occurs by the so called “copy-out paste-in” mechanism. In this case, DNA replication is required to generate an excised transposon circle that is the substrate for the integration step [34]. Replicative transposition leads to proliferation and expansion of TEs, which increases the probability of activating silent AR genes.

## 4. Activation of Silent AR Genes by Promoters Delivered or Generated by TEs

There are various ways in which TEs can enable or modulate the expression of neighboring genes. Some are indirect and involve post-transpositional rearrangement of the genetic architecture (i.e., deletions resulting from homologous recombination between TE copies which bring distant promoters closer to a gene), while others are inherent to the transposition event itself and include (a) derepression of genes, resulting from insertional inactivation of regulatory genes encoding specific transcriptional repressors [35] and (b) promoter delivery. The delivery of promoters is of particular interest in the context of antibiotic resistance since this is the most common phenomenon leading to the activation of silent promotorless genes or increased expression of those with weak promoters [36,37,38,39,40,41].

**Figure 2:**
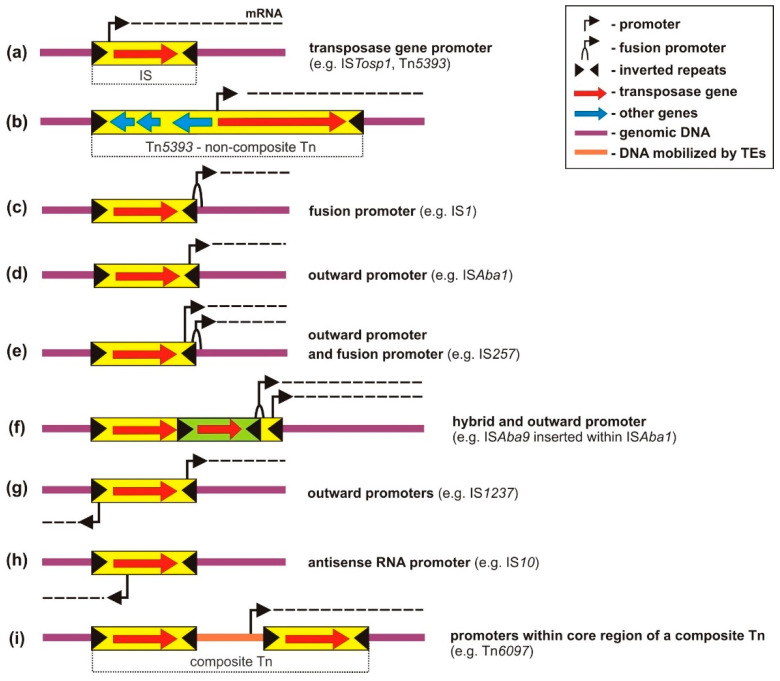
Promoters delivered or generated by TEs that enable or modulate the expression of nearby AR genes: (**a**,**b**) transposase gene promoters; (**c**–**g**) fusion and outward promoters; (**h**) antisense RNA promoters; (**i**) promoters within composite Tns. In the fusion and hybrid promoters, the −35 and −10 hexamers originate from the inserted IS and target DNA, respectively. See text for details.

Only a relatively small number of promoters carried by TEs have been thoroughly characterized [42,43,44]. Although their structure fits into the general promoter scheme, i.e., two short hexameric sequences located at the −10 and −35 positions relative to the transcription start site, it is often difficult to recognize them from their sequence. Most available information on promoter activity has resulted from the analysis of individual transposition events that have led to the emergence of mutated cells with novel phenotypic traits [40,45,46]. More complex studies have been conducted using trap plasmids carrying promoterless AR genes [47]. The diversity of transposition-related events causing the activation of silent genes is astonishing, not in the least due to the involvement of promoters that play distinct roles in the biology of TEs (Figure 2).

### 4.1. Transposase Gene Promoters 

Endogenous promoters of TPase genes usually display weak activity, sometimes even below the detection threshold of the experimental approach used [23]. These promoters are often additionally subjected to tight negative regulation, for example, by inhibitory binding of TE-encoded regulatory proteins or the TPases themselves [48,49]. Multiple regulatory systems, acting at different stages of transposition, help to keep this process in check, which reduces the likelihood of lethality caused by insertional inactivation of housekeeping genes. Consequently, the frequency of transposition of most TEs is low and usually falls within the range from 10^−6^ to 10^−10^ per generation of bacteria [47]. However, this contrasts with metatranscriptomic data from marine microbial communities, demonstrating significant levels of TPase gene expression (up to 2% of all bacterial transcripts) [50]. This study, focused on bacteria of the Baltic Sea, revealed significant differences in TPase expression among IS families (the most highly expressed examples being from the IS*200*/IS*605* family), suggesting differential transcriptional regulation [50]. 

Cotranscription of TPase genes along with those downstream may have been underestimated, since only a few cases have been reported to date. They include (a) several ISs, for example, IS*Tosp1* (IS*200*/IS*605* family) of cyanobacterium *Tolypothrix* sp. [51], IS*Ftu1* (IS*630* family) and IS*Ftu2* (IS*5* family) of *Francisella tularensis* [52] and (b) streptomycin resistance transposon Tn*5393* (Tn*3* family) of *Paracoccus pantotrophus*, found to be able to activate a downstream promoterless tetracycline (ribosome-targeting antibiotic) resistance gene (Figure 2a,b) [47,53]. Tn*5393* is of particular interest because of its frequent transposition (10^−3^, probably directly linked to the strength of the TPase gene promoter), which explains the wide dissemination of this element [and streptomycin (aminoglycoside) resistance] among environmental bacterial isolates [54,55,56].

### 4.2. Fusion Promoters 

A large group of elements (mainly ISs and derivative composite transposons) undergo transposition through circular or dimeric intermediates. The creation of such structures results in the formation of a transient promoter at the IRR-IRL junction (Figure 1c). One of the most thoroughly described examples of this mechanism is the copy-out paste-in transposition pathway of IS*911* (IS*3* family) [57]. IS*911* contains an outward −35 motif in its IRR and a −10 motif in its IRL. Upon formation of a minicircle intermediate, the IRs are joined with a short spacer sequence (3–4 bp, providing optimal spacing) and this newly created fusion promoter drives transcription of a TPase which is necessary for minicircle cleavage and integration of the element into target DNA. The transient promoter is relatively strong (40-fold stronger than the endogenous P_in_ [58], and two-fold stronger than the reference P_lacUV5_ promoter [59]), which ensures that efficient transposition occurs, but only when the intermediate is formed. 

An analogous mechanism is employed by other members of the IS*3* family (IS*2*, IS*LC3*) [60,61], as well as other IS families, including IS*21*, IS*30,* IS*256,* and IS*1111* (elements of this family preferentially transpose into *attC* sites of integrons) [62,63,64,65]. 

The presence of the outwardly directed −35 hexamer within the IRR may facilitate the generation of strong fusion promoters that can drive the transcription of nearby genes. A necessary condition is the presence (or formation upon integration) of a functional −10 promoter motif, correctly spaced in the flanking genomic region (Figure 2c). Increased expression of AR genes resulting from the formation of such a promoter has been demonstrated for numerous ISs representing diverse IS families e.g., IS*1* [66], IS*2* [67], IS*5* [68], IS*6* [69], IS*21* [70], IS*30* [71], IS*256* [72,73], and IS*982* [74]. Several examples are described in Table 1. 

Due to the prerequisite of a sequence at the target site of transposition meeting the criteria of a −10 hexamer, only a limited number of silent AR genes can be activated in this way. However, several studies on target sequence specificity of TEs have shown that some elements preferentially insert into A/T rich DNA regions [75,76]. It has also been observed that a high A/T content of the spacer sequence plays a role in RNA polymerase binding and open complex formation during transcription initiation [77,78]. The results of comparative analyses suggest that some TEs have higher target sequence specificity. For example, an analysis of the distribution of CREE elements (MITE) in *Neisseria* spp. genomes found that the most common direct sequence repeat bordering these elements is a TA duplication, followed by from 3 to 5 bp DRs such as TAT, ATA, TATA, TATAG, or CTATA, resembling the canonical −10 hexamer 5′-TATAAT-3′ [79]. This target sequence preference increases the likelihood of functional fusion promoter formation.

Bacterial promoters are usually identified through their similarities to the consensus *E. coli* promoter sequence (−35 and −10 hexamers: 5′-TTGACA-3′ and 5′-TATAAT-3′, respectively). However, numerous experimental findings have challenged the extent to which this approach is applicable to other bacteria. For instance, promoters of *Bacteroides fragilis* are defined by −33 (5′-TTTG-3′) and −7 (5′-TANNTTTG-3′) regions [95]. This has obvious implications for TE promoter formation, which becomes a host dependent phenomenon, i.e., a particular IS may have the potential to create promoters only in closely related species.

### 4.3. Chimeric TE Promoters 

Several reports have described the generation of novel fusion promoters (illustrating the diversity of TE-generated genetic structures), in which the region containing the −10 hexamer is derived from another TE. These are the so-called chimeric or mosaic promoters (Figure 2f). The genetic environment of *bla*_OXA_ genes in *Acinetobacter baumannii* provides an illuminating case study of such structures. ISs are involved both in dissemination and in expression of carbapenem resistance genes of the *bla*_OXA_ family in *A. baumannii* [96]. One example is the IS*Aba3* element (IS*1* family) (and its variant termed IS*Aba3*-like) commonly associated with the *bla*_OXA-58_ gene, often in the form of an IS*Aba3*-*bla*_OXA-58_-IS*Aba3* platform [97]. IS*Aba3* itself possesses a promoter structure in its IRL which increases the transcription rate of *bla*_OXA-58_ [98]. Moreover, the element is often disrupted by another insertion sequence (most commonly one belonging to the IS*6* family), which frequently results in the formation of a new chimeric promoter, composed of a −35 hexamer located in the IR of the disrupting element and a –10 region in the *tnpA* gene of the then truncated IS*Aba3*(Δ). The integration of another element does not usually abolish the activity of the IS*Aba3*(Δ) complete promoter, and thus, leads to increased expression of downstream genes through an additive (or synergistic) effect of the two promoters. For example, the insertion of IS*Aba825* into IS*Aba3*-like led to from a 32- to a 64-fold increase in the minimum inhibitory concentrations (MICs) of meropenem and imipenem [99]. Transposition of IS*1006* into IS*Aba3*-like resulted in a 12-fold increase in *bla*_OXA-58_ transcription [100]. Analogous chimeric promoters were generated upon insertion of IS*Aba1* (IS*4* family) into IS*Aba9* (IS*982* family) [101], of IS*Aba10* into IS*Aba1* [102], of IS*Aba2* or IS*Aba18* into IS*Aba3* [103], of IS*1008* into IS*Aba3* [89], and of IS*Our1*, IS*1008* or IS*15* into IS*Aba3* [104]. In the final case, the promoter structure was not investigated but the integration of a new element into IS*Aba3* was accompanied by a considerable increase in the MIC for imipenem [89].

### 4.4. Complete Outward-Directed Promoters 

A considerable number of ISs contain complete constitutive outward-oriented promoters in their termini, which can initiate transcription through the IR and into neighboring genes (Figure 2d). Similar to fusion promoters, complete outward-oriented promoters may play an important role in the expression of TPase genes during transposition of ISs via excised circular intermediates (Figure 1c). The occurrence of such promoters has been experimentally confirmed in ISs representing the IS*3* [105], IS*4* [106], IS*5* [42], IS*6* [37], IS*481* [35], IS*982* [42], IS*1380* [29], and IS*L3* [107] families. A prominent example is the element IS*Ecp1* (IS*1380* family), which is responsible for the mobilization for transposition of a number of unrelated antibiotic resistance genes, including several from the *bla*_CTX-M_ groups, encoding a family of extended-spectrum β-lactamases that are dominant worldwide [108]. 

Some ISs possess complete (−35 and −10 hexamers) and also partial (−35 hexamer) outward-oriented promoters (Figure 2e). For example, IS*257* (IS*6* family) was found to drive transcription of a tetracycline resistance gene from a weak complete promoter as well as a strong fusion promoter [37]. A similar situation occurs in the case of the mosaic TE generated by IS*Aba1* and IS*Aba9*, described above (Figure 2f). 

IS*1237* (IS*5* family) is unusual because it contains two active outward promoters at both termini [109] (Figure 2g). These promoters differ in their activity (that at the 3′ end is stronger), but both were shown to drive the transcription of adjacent genes in different Gram-positive bacteria. Thus, IS*1237* is an example of a mobile expression system with a high potential of modulating gene expression, regardless of the orientation in which it is inserted. Several TEs modulating the expression of an adjacent resistance gene by providing a complete outward-directed promoter are described in Table 2. 

### 4.5. Antisense RNA Promoters

Some ISs encode a short RNA fragment which is transcribed from the antisense strand of the TPase gene (asRNA). The annealing of this asRNA to the TPase transcript inhibits its translation thus downregulating transposition. This regulatory mechanism was first described for IS*10*/Tn*10*, where the production of asRNA was controlled by an outwardly directed constitutive promoter, P_out_, located within the first 100 bp from the 5′ end (IRL) of the element [125]. Similar promoters have since been identified in IS*1999* [41], IS*30* [126], and IS*200* [127]. Some P_out_ transcripts can extend into neighboring genes, resulting in their elevated expression (Figure 2h). For IS*10,* this transcription was determined to be around 10% of that produced by reference promoter P_lacUV_ [125]. It is apparent that the localization of these promoters within an element (i.e., how close they are to the IS terminus) is the crucial property influencing their potential to activate adjacent genes by read-through transcription. This would explain why the P_out_ of IS*10* activates adjacent genes, whereas that found within IS*30* (located in its central part; bases 730–760 out of 1200) does not exert any measurable influence on proximal genetic material. Increased expression of AR determinants caused by such promoters has been described for IS*1999* [112] and IS*10* [81].

### 4.6. Promoters Located within Core Regions of Composite Tns 

TEs with a more complex structure (composite Tns and TMos) contain DNA segments of different sizes, often carrying numerous functional passenger genes. If strong, the promoters of these genes may sometimes drive the transcription of genes located close to the site of transposition (Figure 2i). This is the case for a composite transposon Tn*6097* of *Paracoccus ferrooxidans* NCCB 1300066, containing two identical copies of IS*Pfe2* (IS*1634* family) [47]. Insertion of Tn*6097* into trap plasmid pCM132TC caused activation of a downstream promoterless tetracycline resistance gene *tetA*. The promoter responsible for this phenomenon was located in the core region of the element, and the AR gene was cotranscribed with the TPase gene [47] (Figure 2i). 

## 5. Activation of Silent AR Genes from Distant Promoters 

TE-mediated deletions can influence bacterial genomes at higher and lower levels of organization. Large-scale genome reduction (also known as streamlining) accompanies the transition of a free-living organism to an obligate pathogen [128], whereas single deletional events can modify the local transcriptional landscape by removing transcription terminators and generating novel promoter-gene combinations [129]. Such genomic alterations are of special interest in relation to AR, which can emerge if a poorly expressed or silent resistance determinant is brought under the control of a previously distant promoter. Deletions caused by TEs can be categorized according to the underlying molecular mechanism, which depends on the protein machinery involved. This taxonomic approach yields the following classes: (a) homologous recombination, (b) transposition, and (c) transposase-dependent activity of insertion sequence excision enhancer (IEE) protein. 

### 5.1. Deletions Generated by Homologous Recombination 

**Figure 3:**
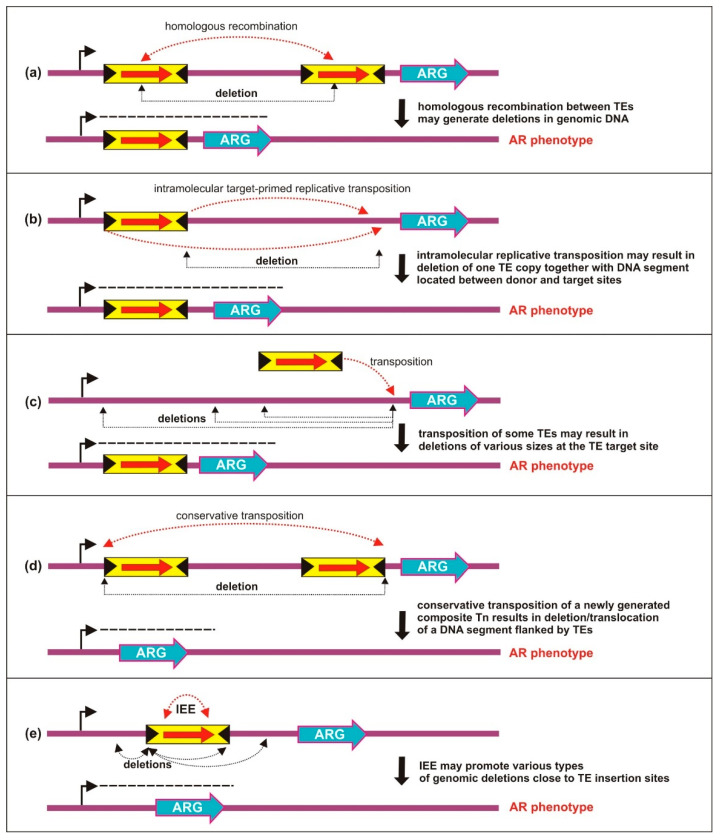
TE-induced deletions leading to activation or modulation of expression of AR genes from distantly located promoters. Deletions generated by (**a**) homologous recombination; (**b**) intramolecular replicative transposition; (**c**) transposition of IS*21*-family members; (**d**) conservative transposition; (**e**) IS excision enhancer protein. ARG—antibiotic resistance gene; IEE—IS excision enhancer protein. Symbols are identical to those in Figure 2. See text for details.

Recombination between homologous directly repeated regions is a common mechanism of deletion formation [130] that naturally occurs where transposons provide portable regions of homology [131]. Being a transposase-independent mechanism (hence, not a feature unique to active TEs) it can also involve MITEs, which in some instances constitute up to 2% of a bacterial genome [79] (Figure 3a). An example of such intramolecular rearrangements is the generation of diverse deletion variants of the *Shigella* spp. virulence plasmid pINV (210 kb), lacking from 40 to 80 kb, produced by ISs acting as “hot spots” for homologous recombination [132].

A more complex case is the deletion resulting from homologous recombination between inversely oriented IS*6110* copies, which normally leads to inversions [133]. Several mechanisms have been proposed, including *recA*-independent recombination between very short homologous sequences (in this case IRs of the IS elements) during DNA replication. This phenomenon of deletion of one of two inverse TE copies and the intervening region has recently been corroborated [134]. The most likely explanation for these deletions was identified as slipped misalignment during replication. 

### 5.2. Deletions Resulting from Transposition of TEs 

Deletions resulting directly from transposition represent a diverse class of events. Various kinds of intramolecular transposition (i.e., transposition at other DNA sites within the same replicon) or activity of an IS excision enhancer protein can lead to the removal of genetic material of variable lengths. A detailed description of the mechanisms involved is beyond the scope of this review; however, below we present a few examples illustrating the enormous diversity of transposition-based phenomena leading to genome rearrangements, which may result in gene activation.

#### 5.2.1. Intramolecular Target-Primed Transposition 

Perhaps the most common deletion of this type is caused by target-primed replicative transposition occurring within the same replicon, which is characteristic of the IS*6* and Tn*3* families. In this reaction, the transposase cleaves the 3′ transposon ends, which can then directly nick the target sequence in two different configurations. An in cis attack leads to deletion of the intervening sequence (which contains the second TE copy) (Figure 3b), whereas an in trans attack causes an inversion [135,136]. Such events represent a case of adjacent deletions [137]; they begin precisely at one of the element’s termini and end at a variable distance in either direction. As the TE-distal endpoint is synonymous with the target insertion site, the deletion size is indirectly determined by the target specificity of a given element. By extension, the size of deletions caused by transposition (and to an extent by homologous recombination) is partly influenced by “target immunity”, a phenomenon that prevents multiple insertions of the element into the same DNA molecule [138]. 

Similar to deletions caused by homologous recombination, the remaining TE lacks DRs since one of the repeats is located within the deleted DNA region. These types of rearrangements are typical for a number of ISs and their derivative composite Tns, such as IS*1*/Tn*9* [135,139], IS*26* [136,140,141], and IS*102* [142], as well as some unit transposons such as Tn*1* (synonymous with Tn*3*) and Tn*103* [143]. The frequency of the rearrangements as well as the ratio of deletions to inversions seem to depend on several conditions, although the exact manner in which they exert their influence is unclear.

#### 5.2.2. Intramolecular Transposition of IS Dimers 

Similar genomic rearrangements may be caused by (IS)_2_ tandems, frequently formed by IS*21* [144] and IS*30* [145]. The deletion occurs when the innermost inverted repeats of two directly repeated head to tail ISs are used to nick the target sequence (in cis orientation). There are two reasons why the reactive junction between the two ISs in a dimer is a highly unstable substrate that readily mediates genomic rearrangements. First, the IRs recognized by the TPase are already in close proximity (resembling the synaptic complex configuration, and virtually identical to the minicircle intermediate) which enables efficient strand cleavage. Second, as already described in the context of TE-borne promoters, the P_junc_ promoter leads to increased transposase expression, enhancing the probability that the reaction will take place. This phenomenon represents a curious case whereby one aspect of transposon biology can have diverse modulatory effects on gene expression, i.e., the potential formation of a hybrid promoter or the promotion of adjacent deletions. Whether other members of the IS*21* and IS*30* families employ a similar mechanism to reorganize DNA is unclear. However, it is highly likely considering the effect associated with the transposition of another IS*21* family member, i.e., IS*Pve1* of *Paracoccus versutus* [47]. Insertion of this element was associated with frequent unidirectional deletions within the insertion site (ranging in size from 0.5 to 4.5 kb), comprising DNA segments adjacent to the 5′ end of the IS [47]. Such IS*Pve1*-induced deletions also led to the activation of a promoterless tetracycline resistance gene *tetA* of trap plasmid pMC132TC (Figure 3c).

#### 5.2.3. Intramolecular Transposition of Composite Tns

In principle, the transposition of any composite Tn via a conservative mechanism can lead to deletions. More precisely, when a genetic region becomes bracketed by two ISs (capable of forming such a transposon), its mobilization can be regarded to be a deletion (Figure 3d). Such events are expected to occur relatively frequently, although few empirical examples have appeared in the literature, with one exception being the detailed report of Watanabe et al. [146]. Once the composite transposon is formed and is moving as a single unit, its transposition is not a deletion in the strict sense because the genetic environment pre-integration and post-excision remains relatively unchanged, although its movement may result in alteration of the local transcription pattern. Deletions can also be caused by “non-canonical” movement of composite transposons as observed in the case of Tn*10*; when the two innermost IRs of ISs (also referred to as inside transposon ends) are used by the TPase, the genetic region between them undergoes deletion (which, as already discussed, would in itself be of special interest only in the case of “primary formation”) and there are two possible pathways for the reaction to follow from that point. In an analogous manner to the aforementioned mechanisms, depending on the relative orientation of the donor and target sequence, the result of such an event is either a deletion (two end products, each segregant containing one IS copy and representing a deletion with respect to the parental genome) or an inversion (one end product with both ISs flanking a region in the reverse orientation) [147].

#### 5.2.4. Deletions Induced by IS Excision Enhancer Protein

An analysis of the transposition of IS*629* (also known as IS*1203v*, IS*3* family) in *E. coli* has led to the discovery that the precise excision of this element (as well as larger deletions involving adjacent regions) is mediated, in a transposase-dependent manner, by a protein named insertion sequence excision enhancer (IEE) [148]. Deletions promoted in this way can be categorized into four types: (a) excision of IS*629* along with the flanking 1–7 bp, (b) excision of IS*629* plus a large adjacent region, (c) partial deletion of IS*629,* and (d) a large adjacent deletion leaving the IS*629* copy intact (Figure 3e). These cover nearly all possible TE-mediated deletion scenarios, with type “b” being potentially the most significant with respect to gene expression because the resulting product lacks a TE copy, which may reduce the distance between a promoter and a downstream gene. The precise molecular mechanism of the IEE-catalyzed reaction is unknown. IEE-mediated excision is now known to occur in members of the IS*3*, IS*1*, and IS*30* families. It is striking that homologs of IEE have been identified in genomes of phylogenetically distant species, their distribution implying spread via horizontal gene transfer. It is probable that the impact of this host-encoded and transposase-dependent excision/deletion system on bacterial genomes is largely underestimated.

## 6. Transient Trans-Replicon Activation of Silent AR Genes 

To investigate transposition-related mechanisms leading to the activation of resistance genes, Dziewit et al. [47] introduced the promoterless *tetA* gene (present in plasmid pCM132TC, containing replication system of a broad-host-range plasmid RK2) into *Paracoccus pantotrophus* strain DSM 11073 (*Alphaproteobacteria*). Under tetracycline selection, several resistant clones were obtained, in which the introduced plasmid pCM132TC was present as part of the cointegrates generated between itself and pKLW1 (approximately 100 kb), a plasmid naturally occurring in strain DSM 11073. 

**Figure 4:**
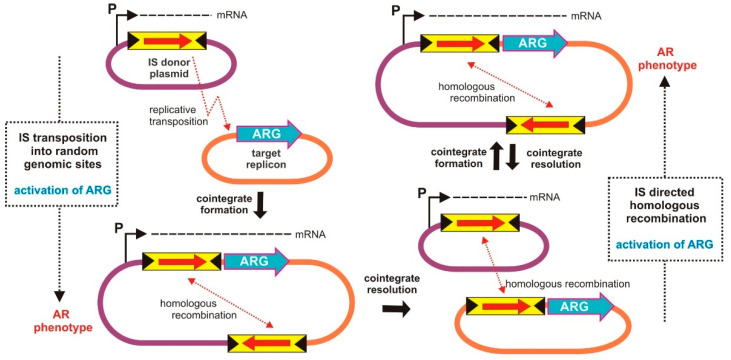
Periodic activation of a transcriptionally silent AR gene by promoters delivered through the formation of cointegrates. ARG—antibiotic resistance gene; *P*—promoter. Replicative transposition of an IS results in formation of a cointegrate between the IS (and promoter) donor and ARG-containing recipient replicons. Homologous recombination leads to resolution of the cointegrate plasmid into two independent IS-containing replicons. Further IS homology directed recombination events lead to the generation of a cointegrate replicon to produce the AR phenotype.

DNA sequencing revealed that the recombinational event that led to the formation of these cointegrates occurred upstream of the *tetA* gene of pCM132TC. The inserted plasmid was bordered by two copies of IS*1248* (IS*5* family), which is a common feature of intermediate forms of replicative transposition (Figure 4). Further analysis revealed that the *tetA* gene was driven by a promoter located within plasmid pKLW1, outside of IS*1248*. 

Resolution of cointegrates into individual plasmids (observed after prolonged growth of the strains under nonselective conditions) resulted in loss of the tetracycline resistance (Tc^r^) phenotype. However, the presence of identical copies of IS*1248* within both plasmids promoted targeted homologous recombination events, resulting in more frequent selection of resistant clones in the presence of tetracycline selection pressure (Figure 4). These observations illustrate the periodic activation of silent AR genes by the transposition-mediated delivery of promoters present in other replicons co-occurring in the cell. 

## 7. Activation of AR Genes Carried by TEs 

In the phenomena described above, the roles of TEs as modulators of gene expression are emphasized, with the particular elements and resistance determinants being largely independent (although their relationships are often associative in nature). These are events of fortuitous promoter delivery or genomic rearrangement leading to the activation (or enhanced expression) of a given gene. The next step in the evolution of the TE–ARG relationship is the establishment of a tighter connection, when a resistance gene becomes a (semi) permanent component of a TE. In such cases, transposons function as transferable platforms promoting the dissemination and expression of AR. 

The more likely transposition is to cause movement of the genes along with the element, the stronger the link between them. Thus, there exists a continuum, with gene mobilizing elements at one end (adjacent DNA sporadically included in the transposing structure) and unit transposons and transporter ISs at the other end (passenger genes contained within these structures can only be lost through events independent of transposition). 

**Figure 5:**
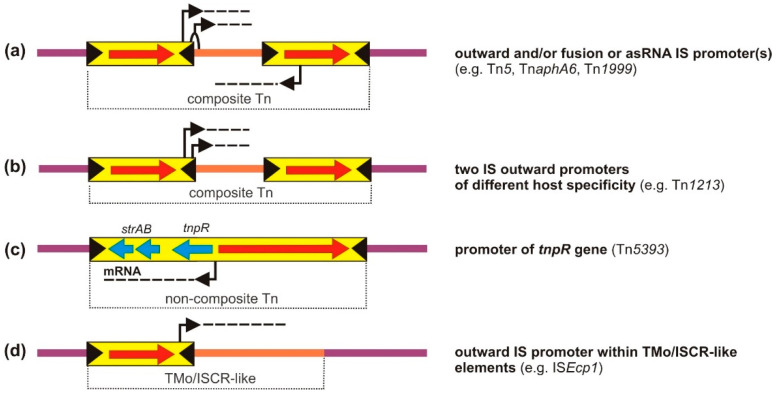
Promoters enabling or modulating the expression of AR genes transferred within Tns: (**a**,**b**) fusion and outward IS promoters within composite Tns; (**c**) internal promoters within non-composite Tns; (**d**) outward IS promoters within TMo/ISCR elements. See text for details. Symbols are identical to those used in Figure 2.

In addition to their role as AR gene disseminators, TEs perform another equally important function as expression systems. Promoters driving the transcription of passenger (or mobilized) genes can originate via one or a combination of several possible scenarios: a structure from the native genetic environment (preceding its inclusion in TE), IS-borne (hybrid or complete), internal promoters of transposon biology modules (transposases or cointegrate resolution systems), or integron promoters (several examples shown in Figure 5). Though conceptually distinct, these two dimensions, transmission and expression, are interlinked in nature. 

### 7.1. Composite Tns

Composite transposons contain a variety of passenger genes and often other TEs within their core regions. At the genome level, these structures are relatively unstable; their “disassembly” (conservative movement of only one IS copy, recombination between the flanking elements, or rearrangements accompanying the non-canonical movement described in the previous section on deletions) is predicted to occur rather frequently. Employing a game theory approach, Wagner [149] confirmed that composite Tns were not an evolutionarily stable strategy at the between genomes level, with one exception, i.e., in the presence of strong selective pressure exerted by antimicrobial compounds. In this regard, composite Tns in which the flanking ISs provide (complete or partial) promoters responsible for core gene expression are of particular interest. There are several examples illustrating the diversity of such strategies. 

The thoroughly studied *E. coli* composite transposon Tn*5* is comprised of two inwardly directed IS*50* elements (IS*50L* and IS*50R*, IS*4* family) flanking determinants for kanamycin (aminoglycoside), bleomycin (glycopeptide), and streptomycin resistance. There is a single nucleotide difference between the ISs, which has important consequences for IS*50L*. This change results in a nonsense mutation, which leads to translation of a truncated, nonfunctional version of the IS*50L* encoded TPase (thus, increasing the likelihood that the structure will mobilize as a single unit) and at the same time produces a more active promoter for the downstream kanamycin resistance determinant. In experimentally constructed variants, where the core region was inverted, and thus, brought under the influence of IS*50R*, the level of transcription was drastically reduced [150].

Other interesting composite transposons include Tn*1999* and Tn*aphA6*. The former is composed of two divergently oriented IS*1999* isoforms (termed IS*1999.2*, IS*4* family) bracketing a *bla*_OXA-48_ gene, encoding a class D β-lactamase. In this case, transcription of the AR gene is driven by a complete P_out_ promoter located in the IS*1999.2*-L, responsible for the generation of asRNA (described in the previous section) [41]. In the latter, an aminoglycoside resistance Tn driven by IS*Aba125* (IS*30* family), the left IS copy, provides the –35 hexamer by replacing the native –35 region of the *aph(3′)-VI* gene, which leads to a four-fold increase in the AR gene expression [151] (Figure 5a).

The insertion of IS*Pa12* and IS*Pa13* (IS*4* family) on both sides of a *bla*_PER-1_ gene (conferring resistance to oxyimino β-lactams) has been shown to lead to the formation of Tn*1213*. IS*Pa12* is responsible for increased transcription of the downstream AR gene. Surprisingly, different sequences within the element are used as promoters depending on the host organism: (5′-TTCAAA)-17N-(TAATCT-3′) in *P. aeruginosa* and (5′-TTCAAA)-16N-(TAAGAA-3′) in *Salmonella enterica* serovar Typhimurium [111] (Figure 5b). This interesting example shows that ISs can contain several “extra” promoters with different host specificities, which may extend the host range of composite Tns they form. 

### 7.2. Non-Composite Tns 

Non-composite Tns (syn. unit- or class II Tns) are intricate modular systems with features highly relevant to AR. Of particular interest is the Tn*3* family of transposable elements which typically contain transposition (*tnpA*, IRs) and cointegrate resolution (*tnpR* or *tnpI* genes and *res* site) modules, plus various types of passenger genes, i.e., AR genes, catabolic operons, and IS elements. Following a fairly recent large-scale study [152], toxin–antitoxin (TA) gene pairs have joined the list of passengers. The various configurations of these building blocks can give rise to portable AR expression platforms.

One notable example is the aforementioned Tn*5393* containing *strA*-*strB* streptomycin resistance determinants (Figure 5c). Through the study of transcriptional fusions, it was established that the resolvase (*tnpR*) promoter located within *res* drives expression of the downstream resistance genes. Expression is repressed by binding of the *tnpR* gene product to this promoter. One identified Tn*5393* genetic variant lacks this repression since it contains an IS*6100* (IS*6* family) insertion within *tnpR*, and therefore, exhibits an elevated level of streptomycin resistance [153,154]. 

It has been proposed that *res*-located promoters control the expression of many passenger genes carried by unit transposons, and this hypothesis has been explored in the case of TA gene pairs [152]. 

Unit transposons containing functional integrons are powerful versatile systems for mobile resistance gene capture. The Tn*21* subfamily of Tn*3* elements represents a paradigm for integron-containing TEs [155]. Integron promoters (P_c_) are known to be functional in a broad range of bacterial species, which permits efficient expression after transfer to a phylogenetically distant host. Expression of the AR gene cassettes is assumed to decline with increasing distance from the P_c_ promoter. However, ISs with the potential to increase expression of resistance genes, regardless of their position relative to P_c_, are often identified within the gene arrays. For example, the presence of IS*Kpn7* (IS*21* family) or IS*Kpn8* (IS*3* family) elements in different genetic variants of the carbapenem resistance Tn*3*-family transposon Tn*4401*, resulted in the formation of strong hybrid promoters and altered expression of the AR gene [91,156]. 

### 7.3. TMos and ISCR-like Elements 

Another class of gene mobilization events involves the transposition of IS*1380* family members (and possibly, in one case, an IS*3* member, IS*Ppy1*, identified in an ancient strain of *Psychrobacter maritimus* preserved in permafrost sediments) [157]. 

An analysis of IS*Pme1* (IS*1380* family) showed that it is capable of mobilizing DNA segments of variable lengths, adjacent to the 3′ end of the element. IS*Pme1* also contains a strong outward-oriented promoter (150-fold stronger than the IS*Pme1* TPase gene promoter) enhancing expression of the mobilized genes in diverse hosts. The structures arising from these gene mobilization events have been termed transposable modules (TMos) [29] (Figure 5d). Another member of the IS*1380* family, IS*Ecp1*, mentioned previously in relation to the dissemination of extended spectrum β-lactamases, gives rise to similar structures. In both cases, it appears that terminal structure misrecognition accompanying regular transposition is responsible for gene capture. More precisely, the transposase can bind to a downstream region resembling the authentic IR, which leads to the inclusion of adjacent material into the transposing structure. Analysis of the variable endpoints of these structures supports this model [29,158]. 

The exact transposition pathway used by these elements is not known. A number of studies have examined the mobilization of genetic material by IS*Ecp1* [120,158,159,160] but, to the best of our knowledge, none of these studies have focused on the fate of the donor site, leaving unanswered the question of whether or not the pathway was conservative. Hybridization patterns of regions containing TMo copies indicate that, in the case of IS*Pme1*, a replicative mechanism is at play [29]. The similarity between the patterns exhibited by the integration of IS*Ecp1* and IS*Pme1*, and the fact that these elements are members of the same IS family, justify their classification as TMos. 

Another efficient gene capture mechanism is displayed by members of an “unusual” IS*91* family (these elements do not generate DRs and their tyrosine transposases are closely related to plasmid replication proteins) [161]. They transpose via processes akin to rolling circle replication (RCR). The first stage involves nicking the single strand for transfer at the 5′ end (*ori91* site) and subsequent replication of the complimentary (non-transferred) strand, which regenerates the donor TE copy. The cleavage of the non-transferred strand at the 3′ end (*ter91* site) leads to the formation of a single-stranded circular intermediate which is later incorporated into a target sequence. Occasionally this second cleavage occurs downstream of the *ter91* site, thus, incorporating flanking DNA into the transposing structure (for IS*1294* the frequency at which this occurs ranges from 1 to 10%) [162]. This mechanism has been further supported by studies on mutants lacking a *ter91* site [163]. As compared with TMos, the IS9*1*-like elements can mobilize much larger DNA fragments (as much as 40 kb in the case of IS*801* [164]). A related group of elements is the insertion sequence common region (common with the *orf* of IS*91* elements), ISCR elements, that are frequently associated with antibiotic resistance genes, and their roles as AR disseminators appear to be non-trivial [165]. 

Although their transposition mechanisms differ, TMos and ISCR-like elements have essentially the same genetic structures and all contain complete outward promoters; both elements represent efficient IS-mediated systems for AR gene capture, expression and spread. 

## 8. Promoter Strength 

Two crucial aspects of a bacterial promoter′s structure influence its strength: (a) the similarity of the –35 and –10 promoter regions (recognized by the sigma subunit of RNA polymerase) to the consensus sequence and (b) the distance between these two hexamers (the optimal length for *E. coli* promoters is 17 bp) [166]. The spacer length is particularly critical, as a one base pair difference can drastically change promoter activity. For example, Jaurin and Normark [67] observed that provision of the –35 region by IS*2* changed the spacer length in one particular promoter from 16 bp to the optimal 17 bp, which resulted in a 20-fold increase in activity, despite low consensus sequence homology of the –35 hexamer. An analogous effect was seen for an IS*256* insertion, where changing the spacer length from 18 to 17 bp led to increased methicillin (narrow-spectrum β-lactam) resistance [72]. The term spacer sequence can also refer to the distance between the promoter and a downstream gene. Ma et al. [167] found that the rate of *bla*_CTX-M_ transcription differed depending on the size of the region separating this AR gene from an upstream IS*Ecp1* copy containing a complete outward-oriented promoter. 

Kamruzzaman et al. [106] analyzed the relative strength of promoters carried by three ISs: IS*Ecp1* (IS*1380* family, responsible for the dissemination and expression of *bla*_CTX-M_ genes); IS*Aba1* (IS*4* family); and IS*Aba125* (IS*30* family, important in the spread and modulation of carbapenem resistance determinants in *A. baumannii*). The relevant properties of the promoters in question (hexamer sequences, spacer length, and match with the *E. coli* consensus) are as follows: IS*Ecp1*—(5′-TTGAAA)-18N-(TACAAT-3′) 5/6, 5/6; IS*Aba1*—(5′-TTAGAA)-17N-(TTATTT-3′) 3/6, 2/6; IS*Aba125*—(5′-TTGAAT-3′) 4/6 (for this study this −35 motif was coupled with a *bla*_NDM-1_ −10 region to generate a fusion promoter). The three IS-borne promoters all had similar effects on the level of expression of a downstream gene (*gfp*); any differences were insignificant in the *E. coli* host, whereas a slight variation was recorded in *K. pneumoniae*. The rate of transcription driven by these ISs was five times greater than the reference P*_tac_* promoter and intermediate between those caused by strong and weak class 1 integron promoter variants.

A related but underexplored topic is the involvement of alternative sigma factors in the expression of genes with upstream TEs. The complete outside promoter within IS*Aba1* contains an extended −10 region (5′-TGACA-3′) upstream of the −10 hexamer (5′-TTATTT-3′). This extended motif (TGn) resembles structures recognized by the σ^S^ subunit of RNA polymerase (RpoS, a central regulator of the general stress response) and is thought to increase the strength of the promoter [44,45]. Notably, the *E. coli* σ^S^ factor “tolerates” deviations from both the consensus sequence and the optimal spacer distance [168]. Since TEs can generate a wide range of variable promoter structures, it is likely that their activity differs depending on the involvement of alternative factors, which could play a role in switching to the stress response under antibiotic pressure. 

## 9. Promoter Fixation 

Increased gene expression or activation of a silent gene caused by a TE-borne promoter is not necessarily permanent. The mobility of TEs coupled with their relatively low rate of transposition can provide a temporary advantage to the host, which may be strongly selected for under certain circumstances. Due to the increasing use of antimicrobial substances, this selective pressure can be so great that some transposons become strongly associated with particular resistance determinants. There are, however, cases where the promoter delivered by a TE becomes a persistent addition to a gene (as persistent as anything in genetics can be). Potron et al. [169] described a large deletion encompassing the majority of an IS*Ecp1* copy upstream of the carbapenemase gene (*bla*_OXA-232_), leaving only ~200 bp of the element, including the complete promoter driving increased transcription of the AR gene. Similarly, Naas et al. [170] identified a remnant of IS*Aba125* (its right-hand end) located upstream of an aminoglycoside phosphotransferase gene (*aphA-6*). This fragment contained a −35 hexamer and together with a −10 region (outside the element) it provided a fusion promoter which was likely responsible for expression of this AR gene that led to amikacin (aminoglycoside) resistance in the studied strain of *P. aeruginosa* [170]. In the aforementioned cases, the transition of a promoter-containing TE to a non-autonomous state can be viewed as a fixation event that has stabilized the linkage between the promoter and the AR gene. The contribution of vestigial elements, once part of distinct TEs, to gene expression is probably underestimated because transposon remnants pervade genomic sequences deposited in public repositories.

## 10. Conclusions

This review provides evidence for the significant influence of TEs (complete or partial) on AR gene expression. The multitude of different strategies employed, and the emerging transposon-based expression systems are astonishing. Given the widespread abundance of TEs in bacterial genomes and their strong association with AR genes [171], there is no doubt that these elements play key roles both in creating and in disseminating diverse resistance phenotypes. 

Antibiotics impose strong selection pressure for the resistance. Interestingly, it has been shown that some of these compounds can also lead to an increase in the transpositional activities of certain TEs. For instance, the transposition frequency of IS*256* in *S. aureus* cells was increased four-fold in the presence of subinhibitory concentrations of chloramphenicol, linezolid, and spectinomycin (relevant in human and veterinary medicine) [172], which may accelerate the development of resistance phenotypes.

Numerous studies have focused on the characterization of individual TEs and the recombination events they determine. Such events leading to AR can be readily recognized by positive antibiotic selection. However, in order to determine the full impact of TE-dependent changes on bacterial transcriptomes, targeted analyses of global omics are necessary. It is equally important to understand the molecular basis of the functioning of a much larger pool of TEs, representing diverse families of elements. Greater knowledge of their biology and specific properties may permit the identification of novel pathways and mechanisms leading to the activation of silent genes. 

## Figures and Tables

**Table 1:** AR gene activation caused by TE-born fusion promoters.

IS/Tn Family	Element	Resulting Resistance to:	Gene	Organism	Reference
IS*1*	IS*1*	ampicillin	*bla*_TEM-1_	*E. coli*	[80]
		fluoroquinolones (marbofloxacin, enrofloxacin, ciprofloxacin), florfenicol, erythromycin	*acrEF*	*Salmonella enterica*	[81]
	IS*1*-like	ceftazidime, aztreonam	*bla*_TEM-6_	*E. coli*	[66]
IS*3*	IS*2*	kanamycin	*neoR*	*E. coli*	[82]
		erythromycin, clarithromycin, azithromycin, clindamycin, linezolid	*acrEF*	*E. coli*	[83]
		ampicillin	*ampC*	*E. coli*	[67]
IS*6*	IS*26*	β-lactams (amoxicillin, ticarcillin, piperacillin, cephalothin, cefoxitin, ceftazidime, cefotaxime, aztreonam)	*bla*_SHV-2a_	*Pseudomonas aeruginosa*	[84]
		gentamicin	*aacC3*	*E. coli*	[85]
		β-lactams (penicillin, cefotaxime, aztreonam)	*bla*_BES-1_	*Serratia marcescens*	[86]
		neomycin, kanamycin, paromomycin, lividomycin	*aphA7*	*Klebsiella pneumoniae*	[87]
	IS*Our1*	carbenicillin	*bla*_ABA-1_	*Oligella urethralis*	[88]
	IS*1008*	carbapenem	*bla*_OXA-58_	*Acinetobacter baumannii*	[89]
	IS*257*	tetracycline	*tetA(K)*	*Staphylococcus aureus*	[37]
		trimethoprim	*thyE-dfrA-orf140*	*S. aureus*	[90]
IS*21*	IS*Kpn7*	imipenem	*bla*_KPC_	*K. pneumoniae*	[91]
	IS*Bf1* (IS*21*-like)	ampicillin	*cepA*	*Bacteroides fragilis*	[70]
IS*30*	IS*18*	aminoglycosides (amikacin, netilmicin, tobramycin)	*aac(6′)-Ij*	*Acinetobacter* sp. 13 strain BM2716	[92]
	IS*Aba125*	cephalosporin	*bla*_ADC_	*A. baumannii*	[93]
IS*256*	IS*256*	methicillin	*mecA*	*Staphylococcus sciuri*	[73]
			*llm*	*S. aureus*	[72]
Tn*3*	Tn*3*	gentamicin	*aacC2*	*E. coli*	[94]

**Table 2:** AR gene activation caused by TE-borne complete outward-oriented promoters.

IS/Tn Family	Element	Resulting Resistance to:	Gene	Organism	Reference
IS*4*	IS*Aba1*	ceftazidime	*ampC*	*A. baumannii*	[40,45]
		β-lactams (ticarcillin, piperacillin, aztreonam)			
		cephalosporins (cefuroxime, cefoxitin, cefotaxime, ceftazidime, cephalothin)	*ampC*	*A. baumannii*	[44]
		carbapenem	*bla*_OXA-23_	*A. baumannii*	[110]
		cephalosporins (ceftazidime, cefepime), gatifloxacin)	*bla*_ADC_	*A. baumannii*	[93]
	IS*Pa12*	β-lactams (amoxicillin, ticarcillin, piperacillin, cefuroxime, ceftazidime, cefotaxime, cefepime, aztreonam)	*bla*_PER-1_	*S. enterica*	[111]
		(amoxicillin, ticarcillin, cefuroxime, ceftazidime, cefotaxime, cefepime, aztreonam)		*P. aeruginosa*	[111]
	IS*10*	fluoroquinolones (marbofloxacin, enrofloxacin, ciprofloxacin), florfenicol, erythromycin	*acrEF*	*S. enterica*	[81]
	IS*1999*	ceftazidime	*bla*_OXA-48_	*K. pneumoniae*	[41]
			*bla*_VEB-1_	*P. aeruginosa*	[112]
IS*5*	IS*1168*	5-nitroimidazole	*nimA, nimB*	*Bacteroides*	[39,113]
	IS*1169*	5-nitroimidazole	*nimD*	*B. fragilis*	[114]
	IS*1186*	carbapenem	*cfiA*	*B. fragilis*	[36]
IS*6*	IS*257*	tetracycline	*tetA(K)*	*S. aureus*	[37]
	IS*1006*	imipenem, meropenem	*bla*_OXA-58_	*A. baumannii*	[115]
	IS*1008*	imipenem, meropenem	*bla*_OXA-58_	*A. baumannii*	[115]
IS*30*	IS*4351*	tetracycline, chloramphenicol		*B. fragilis*	[116]
IS*982*	IS*1187*	carbapenem	*cfiA*	*B. fragilis*	[42]
	IS*Aba4*	carbapenem	*bla* *_OXA-23_*	*A. baumannii*	[110]
	IS*Aba825*	carbapenem	*bla*_OXA-58-like_ *, bla*_OXA-65_	A. *baumannii*	[117]
IS*1380*	IS*Ecp1*	β-lactams (amoxicillin, ticarcillin, piperacillin, cephalothin, cefoxitin, ceftazidime, cefotaxime, cefpirome, aztreonam)	*bla*_CTX-M-15_	*Enterobacteriaceae* (*E. coli, K.* *pneumoniae, Enterobacter aerogenes*)	[118]
		β-lactams (amoxicillin, piperacillin, cephalothin, cefuroxime, cefotaxime, aztreonam)	*bla*_CTX-M-17_	*K. pneumoniae*	[119]
		β-lactams	*bla*_CTX-M-19_	*K. pneumoniae*	[120]
		cefotaxime	*bla*_CTX-M-2_	*Kluyvera ascorbata*	[121]
		extended-spectrum cephalosporin (cephalothin, cefpodoxime, cefotaxime, ceftazidime, cefmetazole), ampicillin, aztreonam	*bla*_CMY-4_	*K. pneumoniae*	[122]
		aminoglycosides (gentamicin, streptomycin)	*rmtC*	*E. coli*	[46]
	IS*612*	imipenem	*cfiA*	*B. fragilis*	[43]
	IS*613*	imipenem	*cfiA*	*B. fragilis*	[43]
	IS*613*-like	imipenem	*cfiA*	*B. fragilis*	[123]
	IS*614*	imipenem	*cfiA*	*B. fragilis*	[43]
	IS*615*	imipenem	*cfiA*	*B. fragilis*	[43]
	IS*616*	imipenem	*cfiA*	*B. fragilis*	[43]
	IS*942*	imipenem	*cfiA*	*B. fragilis*	[42]
	IS*943*	imipenem	*cfiA*	*B. fragilis*	[124]
	IS*1188*	carbapenem	*cfiA*	*B. fragilis*	[42]
